# Piggyback-the-Winner in host-associated microbial communities

**DOI:** 10.1038/npjbiofilms.2016.10

**Published:** 2016-07-06

**Authors:** Cynthia B Silveira, Forest L Rohwer

**Affiliations:** 1 Department of Biology, San Diego State University, San Diego, California, USA

## Abstract

Phages can exploit their bacterial hosts by lytic infection, when many viral particles are released at cell lysis, or by lysogeny, when phages integrate into the host’s genome. We recently proposed a new dynamic model of bacteria–phage interactions in which lysogeny predominates at high microbial abundance and growth rates. This model, named Piggyback-the-Winner (PtW), contrasts to current accepted models on the frequency of lysis and lysogeny and predicts that phages integrate into their hosts’ genomes as prophages when microbial abundances and growth rates are high. According to PtW, switching to the temperate life cycle reduces phage predation control on bacterial abundance and confers superinfection exclusion, preventing that a closely-related phage infects the same bacterial cell. Here we examine how PtW is important for metazoans. Specifically, we postulate that PtW and the recently described bacteriophage adherence to mucus (BAM) model are strongly interrelated and have an important role in the development of the microbiome. In BAM, phage produced by the microbiome attach to mucins and protect underlying epithelial cells from invading bacteria. Spatial structuring of the mucus creates a gradient of phage replication strategies consistent with PtW. We predict that lysogeny is favored at the top mucosal layer and lytic predation predominates in the bacteria-sparse intermediary layers. The lysogeny confers competitive advantage to commensals against niche invasion and the lytic infection eliminates potential pathogens from deeper mucus layers.

## Introduction

Lytic and temperate phage infections have markedly different outcomes for the bacterial host. In the former, phage act as predators and control bacterial abundance via cell lysis. Lytic predation has been modelled according to classical predator–prey Kill-the-Winner dynamics, in which a fast-growing bacterial strain is killed by its phage until another strain rises, leading to control of bacterial community abundance.^1^ In contrast, during a temperate infection, the phage integrates into the host chromosome, replicating with their host as a prophage (some phages can replicate as plasmids). Prophage protect the host cell from infection by closely related phage via superinfection exclusion, a process in which various proteins block other phage from establishing a productive infection. Prophage also confer fitness improvement via lysogenic conversion, i.e., prophage expresses genes that alter the lysogen's physiology.^2^ The lytic to lysogenic switch has been characterised in a small number of host-phage models including *E. coli* and lambda phage, however, the factors determining the fate of phage infections in complex microbial communities remains poorly understood.

The oceans are currently the most extensively explored biome in regards to phage–bacteria interactions.^3^ Studies based on prophage induction by DNA-damaging agents suggest that lytic infections are more common in high nutrient availability conditions, whereas lysogeny is more frequent in oligotrophic conditions.^4,5^ Recently, using a combination of approaches including the analysis of bacterial and viral counts, metagenomics and experimental manipulation of host growth rates we demonstrated that lysogeny is in fact a successful strategy when bacteria are in high abundance and growing rapidly.^6^ Host availability and metabolic status are important factors that determine whether phage infection occurs via lytic dynamics or lysogeny. Coral reefs worldwide are currently experiencing microbialization, a phenomenon defined as the increased abundance and energetic demands of microbes in relation to that of macrobes (e.g., fish) as a result of anthropogenic disturbance.^7,8^ Therefore, coral reefs represent an exceptionally useful natural system for the study of lysis/lysogeny decisions in complex microbial communities. We observed a negative relationship between the virus-to-microbe ratio (VMR) and bacterial abundance in coral reefs across the Pacific and Atlantic Oceans spanning a gradient of human impact, showing that at high bacterial abundances there were relatively fewer phage. High VMR is observed when lytic predation is high, thus the negative relationship we observed was the first indication of a decreased importance of lysis in high bacterial abundance environments. Metagenomic analysis showed that the rise of resistant bacterial strains does not explain the decrease in lytic production, a mechanism proposed based on Red Queen co-evolutionary dynamics.^9^ Instead, viral metagenomes isolated when bacterial abundance was high showed an increased representation of integrase and excisionase genes, hallmarks of temperate phage, along with prophage-like sequences, demonstrating that lysogeny is more predominant at high bacterial abundances. By experimentally manipulating bacterial community growth rates in laboratory conditions, we demonstrated that high bacterial growth is the driver of the switch to lysogeny. This transition was later corroborated by the analysis of 2110 bacterial genomes, showing that minimal doubling time is the life-history trait mostly correlated with the frequency of prophages.^10^

On coral reefs, phage lysogenic switches enhance ecosystem microbialisation modifying microbial interactions in the food web.^11^ In this scenario, phage control of bacterial abundance through lytic infection is impaired, releasing phage predation control of bacteria. Added to the growth stimulus of high dissolved organic carbon release from algae in degraded reefs, PtW dynamics contributes to increased bacterial biomass and energetic requirements.^7^ As water-column microbes are implicated in particulate matter formation and benthic pelagic coupling, microbialization increases the flux of carbon back to the benthic community, reshaping trophic relationships.^11,12^

## How does Piggyback-the-Winner work in mucosal surfaces?

Metazoans harbour complex microbial communities that function together forming holobionts.^13^ Mucosal surfaces are hotspots of microbial colonisation in metazoan hosts such as in coral mucus and the human gut and lungs.^14,15^ Microbial abundance in coral mucus can be be as high as 1.8×10^6^ cells per ml, which is almost an order of magnitude higher than the overlying water.^16^ Phages are diverse and abundant members of metazoan mucosal communities^17,18^ and exhibit shifts in community composition during environmental perturbations or the rise of diseased states in their host.^19,20^ Few studies have tried to determine the abundance of phages in mucosal surfaces. Barr *et al*.^21^ showed that VMR is in average four times higher in metazoan-associated mucosal surfaces when compared with the surrounding environment. Phages can directly interact with metazoan mucins through the expression of proteins with immunoglobulin-like domain on their capsids.^21^ By attaching to mucins, phage display sub-diffusive hunting behaviour (i.e., move slower than would be expected by diffusion), enhancing their abilities to find, infect and kill bacteria in the mucus environment by increasing the rate of phage–bacteria encounters.^22^ This model of bacteria–phage interaction in holobionts is called bacteriophage adherence to mucus (BAM) and highlights the existence of a phage-mediated form of immunity.^23^ High rates of lytic infection in bacteria-rich mucosal surfaces contrast to predictions of PtW, since lysis is expected to be reduced in these conditions. Metagenomic analysis of mucosal viral communities from the human gut and lungs show these communities have strong lysogenic signatures.^24,25^ It is estimated that up to 50% of viral particles in the human gut are temperate, though the BAM model lacks predictions about the role of lysogeny in mucus. Although lysogenic infections may fit into the BAM model as a form of memory against invasive bacterial strains, high microbial abundances and VMR in these environments makes it challenging to reconcile these two well-characterised phage–bacteria interaction dynamics in holobionts. While it is known that these two dynamics occur, their conditions and outcomes are remarkably different. Here we reconcile the two models based on mucus spatial structure.

## Spatial structure determines lysis/lysogeny switches in holobionts

**Figure 1:**
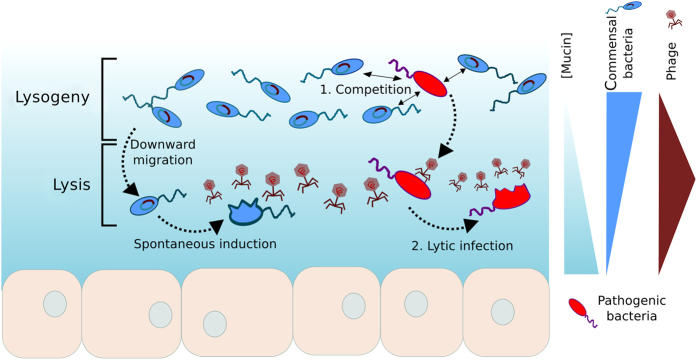
Spatially structured lytic to lysogenic switches in microbial communities associated to metazoan mucosal surfaces. The bottom nucleated cell layer represents the metazoan host epithelium. A mucin concentration gradient is formed from the epithelium to the top. Commensal bacteria abundance (blue flagellated cells) is higher at the top layer where mucin concentration is low, whereas phage abundance is higher at intermediate layers as a result to direct adherence to mucus. High bacterial abundance and growth at the top layers favours lysogeny, while lysis is favoured at the intermediate layer. An invading pathogen (red flagellated cell) encounter two steps of phage-mediated immunity: (1) competition with commensal lysogens displaying high fitness resulting from the integrated phage genome; (2) lytic infection at intermediate layers. An invasive lysogen containing a prophage that confers high fitness gain could outcompete commensals and scape lytic infection by maintaining high growth rates, leading to the establishment of infection.

Metazoan mucosal surfaces display well-defined spatial organisation. Constant secretion of mucin molecules by epithelial cells creates a mucin concentration gradient, with high concentrations of mucins in the inner layers close to the epithelium and lower concentrations in the outer layers, where mucus is continually sloughed out.^26^ The dynamic nature and tridimensional complexity of mucus are reflected in gradients of microbial colonisation found in vertical profiles of mucus layers. Bacterial abundance is higher in the outer layer, whereas the inner layer is virtually deprived of bacterial cells.^27,28^ As a result, bacterial densities display the opposite gradient of that observed for mucins. Phage distribution in mucosal surfaces is predicted to be positively correlated with mucin concentration in outer and mid mucus layers, given the direct interaction between phage capsids and mucin molecules, and then decreases closer to the epithelium.^21,22^ Thus, VMR is higher at intermediate mucin concentration found in the middle of the mucus layer (Figure 1).

In order to make predictions about where lysis and lysogeny will be favoured in the tridimensional structure of the mucus, it is also important to consider the distribution of bacterial growth rates in the mucosal layers. There is evidence that bacterial growth rates are higher in top layers of biofilm, where we also find higher bacterial abundance.^29,30^ In contrast to free-living communities, nutrients are not a limiting factor in the rich-nutrient environment of metazoan mucus. In internal mucosal surfaces, such as human lungs and gut, bacterial growth rates in deeper mucosal layers are primarily limited by oxygen availability, similar to tridimensional profiles of biofilm communities growing on non-biological surfaces.^31,32^ Independent of electron acceptor availability, dehydration of deep mucosal layers because of high mucin concentration is a strong growth limitation, making this area virtually microbial-free.^29,33,34^ High mucin concentration creates nanoscale pores and increases mucus viscoelasticity, impairing bacterial penetration and growth.^35^ In these conditions, bacteria tend to form aggregates on the top layers the mucosal surface and the resulting gradient in bacterial growth rate profiles is similar to that of bacterial abundance (Figure 1).

Piggyback-the-Winner dynamics predict that lysogeny will be favoured in the top of the mucus layer, where bacterial abundance and growth rates are higher. Therefore, most of the commensal bacteria would host a prophage in their genomes and be protected against superinfection.^2^ As a lysogenic bacteria moves downward in the mucin gradient and approaches the epithelium, bacterial growth rates and density decreases, favouring prophage induction and cell burst. Cell lysis and release of phage progeny in turn contributes to the maintenance of high VMR in intermediary mucin layers. From this dynamic emerges a spatial organisation of phage replication strategies in which lysogeny is dominant on the top mucus layers while lysis occurs mainly at the intermediary mucus layers (Figure 1). This model of lysis/lysogeny switch reconciles the two recently proposed mechanisms of virus–bacteria interaction in holobionts.

By situating phage replication strategies in the complex tridimensional structures of mucosal surfaces we are able to further develop the proposed phage-mediated immunity mechanism.^23^ An invasive bacterium arriving to a host-associated microbial community would face two consecutive steps of protection by the commensal community: the first is the competition with the commensal microbes for resources at the top layer, where commensal bacteria fitness is high as a result of high rates of lateral gene transfer by transduction in phage integration. If this invasive bacterium passes this first line of defense, then high phage abundance at the deeper layers of the mucin gradient will increase the chance of viral infection. Assuming this microbe has no previous immunity to the commensal phage strains and infection occurs, growth conditions at the deeper mucin layer will favour lytic infection, eliminating the invasive bacterium. Two levels of phage-mediated immunity arise from this model: (1) widespread lysogeny at the top mucus layers protects commensal microbes from superinfection exclusion and increases their fitness; and (2) phage infection eliminates the invasive pathogens in deeper mucus though lysis, protecting the metazoan host against invasive pathogenic bacteria.

## Lysogeny and the co-evolution of bacteria and phage in mucosal surfaces

Although integration of viral genome into the bacterial chromosome has beneficial effects for the host through superinfection exclusion and lateral gene transfer, it is also argued that it represents an extra energetic expense, reducing host fitness.^36^ Energetic costs resulting from resistance to infection through CRISPRs and mutation of membrane receptors lead to a trade-off between competition and resistance.^37^ In models of virus–bacteria interaction this trade-off predicts that resistant bacterial strains display lower growth rates compared with sensitive ones.^9,38^ However, in nutrient-rich conditions, such as the mucosal surface environment, energetic costs of carrying a prophage would be reduced or insignificant, and host fitness can actually be improved through acquisition of useful genes.^2,39^ Superinfection exclusion is also a more efficient mechanism for escaping lysis than resistance through mutation, thereby avoiding Muller ratchet-like declines in fitness.^40^ Red Queen dynamics predicts that antagonistic evolution of lytic phages and bacteria increases molecular evolution in both predator and prey.^41^ In a PtW scenario, gain of genomic islands formed by prophages would be a more important diversification mechanism than the molecular evolution of CRISPRs elements, membrane receptors and competence genes.^41,42^

In the spatially structured environment of mucosal surfaces, high lytic activity in intermediary layers, where phage display sub-diffusive motion increasing chances of host encounter, is predicted to increase diversification through antagonistic evolutionary mechanisms.^22,43^ Conversely, the main diversification mechanisms in the outer layers, where lysogeny is more prevalent, are lateral gene transfers and genomic islands created by phage integration.^2^ In contrast to homogenous environments, spatial gradients in lysis and lysogeny in mucosal surfaces are predicted to contribute to maintenance of steady-state community assembly though spatial refuges and coexistence stabilization.^44,45^

## Implications of Piggyback-the-Winner for polymicrobial diseases

Many metazoan diseases are characterised by the colonisation of mucosal surfaces by complex and dense microbial communities that differ from the healthy microbiome.^46,47^ Cystic fibrosis (CF) is a the genetic disease caused by mutations in an ion chloride channel that leads to cationic influx and dehydration, compromising the ability of cells to clear (i.e., remove) mucus.^48,49^ In CF lungs, the thick, persistent mucus layer facilitates chronic microbial colonisation that have been extensively studied and will be explored here as a model for PtW dynamics in polymicrobial infections.^50–52^ Viruses are abundant members of the CF microbial community and encode genes related to clinically important microbial phenotypes, including small colony variants and antibiotic resistance.^53,54^ High microbial abundances and growth rates in CF mucus are predicted to stimulate PtW dynamics. In extreme, advanced CF states phages can be virtually absent from microbial biofilms, indicating bacterial escape from top–down control through lysogeny.^53^

In chronic infections, PtW dynamics have the potential to benefit the long-term opportunistic pathogens instead of commensal microbes, representing a threat for the human host. The relative abundance of free temperate phages capable of lysogenic conversion of the liverpool epidemic strain of *Pseudomonas aeruginosa*, a common CF opportunistic pathogen, decreases with increasing *P. aeruginosa* abundance in the lung.^55^ This negative relationship observed though quantitative PCR targeting specific phage strains is identical to that observed for whole community analysis in marine environments, soils, freshwater and so on.^6^ VRM reduction during *P. aeruginosa* blooms indicates higher integration rates during periods of high bacterial growth rates, consistent with PtW dynamics. From these observations, we predict that increased lateral gene transfer of virulence factors and antibiotic resistance genes resulting from high integration rates will enhance pathogen fitness. Lysogenic conversion of liverpool epidemic strain *P. aeruginosa* by PAO1 phages results in high *P. aeruginosa* competitive advantage in CF mouse model, and transposon mutagenesis of liverpool epidemic strain prophages reduces bacterial competitiveness, corroborating our predictions.^56,57^

The dual role of PtW in mucosal surfaces, either creating a phage-mediated immune mechanism or enhancing disease states, as in CF, will be determined by initial mucosal colonisation and interference by external factors such as administration of antibiotics. Invasion of commensal communities by a pathogen harbouring a prophage with a large host spectrum would facilitate pathogenic take-over by (a) increasing the fitness and competitive advantage of the pathogen over resident microbes, allowing the pathogen to surpass the first level of immunity in Figure 1, and (b) killing through lytic infection the commensal bacteria that display lower growth rates compared with the pathogen. CF lung colonisation by opportunistic pathogens occur in early ages, when patients are often receiving antibiotics.^58^ These drugs suppress the resident microbial community, opening niches for pathogenic lysogens and leading to the establishment of their chronicle infections.^59^ *Staphyloccocus aureus* infections have a pivotal role in early stages of CF lung injury, which lead to the proposal of prevention of *S. aureus* infection in children.^60^ Although anti-staphilococcal prophylaxis in infants and young children with CF suppresses *S. aureus* colonisation, it increases the likelihood of *P. aeruginosa* infection over the course of a 5 to 7 years treatment.^61^ Early-life antibiotics treatment sets the stage for the long-term establishment of opportunistic pathogens carrying prophages encoding antibiotic resistance and virulence genes.^62^ Changes in antibiotics treatment and the addition of multiple drugs over the patient’s life span have the potential to trigger expression of virulence factors and lead to CF exacerbation events.^63,64^

## Future directions

Various culture-based and metagenomics studies cited here provide strong evidence for our model of PtW dynamics in spatially structured environments such as mucosal surfaces. However, further studies that test this model experimentally are necessary in order to fully understand its underlying controls and provide tools for how it may be applied in clinical settings. The first question to be answered is the profile of bacterial growth rates on mucus gradient, and if the growth rate profiles are consistent in various model systems. The use of single-cell resolution confocal microscopy, as applied before on *Vibrio cholera* biofilms, can show the architectural separation of bacterial growth, helping to understand growth rate constraints in mucosal surfaces^66^. The next fundamental step is to quantify the frequency of lysogenic cells across the mucosal surface. To avoid the well-known bias of prophage induction assays, the application of high resolution microscopy and genome sequencing are the most advanced approaches available. The use of optical tweezers to isolate individual cells followed by large scale genome sequencing of single cells can provide reliable information on the frequency of lysogeny across mucus layers and the diversity of integrated prophages.^67,68^

An intriguing aspect arising from the interplay between PtW and BAM is the dual role of PtW in shaping the healthy microbiome and, alternatively, facilitating the establishment of polymicrobial diseases. To understand these processes, the application of microfluidic devices can be scaled up to complex communities and essays with engineered lytic and temperate phages, shedding light on the success rates of lytic and lysogenic infections^22^. A microfluidic flow device applied to the study of coral micropropagates, called coral-on-a-chip, could allow researchers to track the onset of microbial colonization, fitness gain, and lateral gene transfer on coral mucosal surfaces^65^. Finally, the role of PtW and BAM in the context of disease states will need to be addressed with the use of *in vivo* models. High-throughput quantification of microbial organization in the gut of gnotobiotic and human microbiota-colonized mice recently demonstrated that diet alters mucus thickness in the distal colon, increasing proximity of microbes to the epithelium^27^. Immunofluorescence tracking of bacteria and phage using this pipeline can improve understanding of the effect of bacterial migration through the mucus framework on phage integration and induction.

## Concluding remarks

Here we reconcile two recently proposed models of bacteria–phage dynamics, PtW and BAM in host-associated microbiomes. We propose a spatially defined model where PtW is more prevalent in outer layers of mucosal surfaces where microbial abundance and growth rates are high. In intermediate layers, high virus-to-microbe ratios facilitated by BAM and phage sub-diffusive motion favour lytic dynamics. A two-level phage-mediated immunity mechanism arises from this model: (1) bacterial lysogens at the top mucus layers have a competitive advantage against invasive pathogens; and (2) a pathogen that reaches deeper layers is attacked by lytic infection. Invasion by a lysogenic pathogen could, on the contrary, benefit the pathogen, enabling the onset of persistent infections.

## References

[bib1] Thingstad, T. F. Elements of a theory for the mechanisms controlling abundance, diversity, and biogeochemical role of lytic bacterial viruses in aquatic systems. Limnol. Oceanogr. 45, 1320–1328 (2000).

[bib2] Obeng N., Pratama A. A. & van Elsas J. D. The significance of mutualistic phages for bacterial ecology and evolution. Trends Microbiol. 24: 440–449. (2016). 26826796 10.1016/j.tim.2015.12.009

[bib3] Rohwer, F. & Thurber, R. V. Viruses manipulate the marine environment. Nature 459, 207–212 (2009). 19444207 10.1038/nature08060

[bib4] Paul, J. H. Prophages in marine bacteria: dangerous molecular time bombs or the key to survival in the seas? ISME J. 2, 579–589 (2008). 18521076 10.1038/ismej.2008.35

[bib5] Paul, J. H. & Jiang, S. C. Occurrence of lysogenic bacteria in marine microbial communities as determined by prophage induction. Mar. Ecol. Prog. Ser. 142, 27–38 (1996).

[bib6] Knowles, B. et al. Lytic to temperate switching of viral communities. Nature 531, 466–470 (2016). 26982729 10.1038/nature17193

[bib7] McDole, T. et al. Assessing coral reefs on a Pacific-wide scale using the microbialization score. PLoS ONE 7, e432331–10 (2012). 10.1371/journal.pone.0043233 PMC3436891 22970122

[bib8] Haas, A. F. et al. Global microbialization of coral reefs. Nat. Microbiol. 1 (2016). 10.1038/nmicrobiol.2016.42 27572833

[bib9] Thingstad, T. F., Våge, S., Storesund, J. E., Sandaa, R.-A. & Giske, J. A theoretical analysis of how strain-specific viruses can control microbial species diversity. Proc. Natl. Acad. Sci. USA 111, 7813–7818 (2014). 24825894 10.1073/pnas.1400909111 PMC4040589

[bib10] Touchon, M., Bernheim, A. & Rocha, E. P. C. Genetic and life-history traits associated with the distribution of prophages in bacteria. ISME J. (e-pub ahead of print 25 March 2016; doi:10.1038/ismej.2016.47). 10.1038/ismej.2016.47 PMC5113838 27015004

[bib11] Silveira, C. et al. Microbial and sponge loops modify fish production in phase-shifting coral reefs. Environ. Microbiol. 17, 3832–3846 (2015). 25817914 10.1111/1462-2920.12851

[bib12] de Goeij, J. M. et al. Surviving in a marine desert: the sponge loop retains resources within coral reefs. Science 342, 108–110 (2013). 24092742 10.1126/science.1241981

[bib13] Knowlton, N. & Rohwer, F. Multispecies microbial mutualisms on coral reefs: the host as a habitat. Am. Nat. 162, S51–S62 (2003). 14583857 10.1086/378684

[bib14] Rohwer, F., Seguritan, V., Azam, F. & Knowlton, N. Diversity and distribution of coral-associated bacteria. Mar. Ecol. Prog. Ser. 243, 1–10 (2002).

[bib15] Huttenhower, C. et al. Structure, function and diversity of the healthy human microbiome. Nature 486, 207–214 (2012). 22699609 10.1038/nature11234 PMC3564958

[bib16] Garren, M. & Azam, F. New method for counting bacteria associated with coral mucus. Appl. Environ. Microbiol. 76, 6128–6133 (2010). 20656857 10.1128/AEM.01100-10 PMC2937480

[bib17] Breitbart, M. et al. Metagenomic analyses of an uncultured viral community from human feces. J. Bacteriol. 185, 6220–6223 (2003). 14526037 10.1128/JB.185.20.6220-6223.2003 PMC225035

[bib18] Vega Thurber, R. L. et al. Metagenomic analysis indicates that stressors induce production of herpes-like viruses in the coral *Porites compressa*. Proc. Natl Acad. Sci. USA 105, 18413–18418 (2008). 19017800 10.1073/pnas.0808985105 PMC2584576

[bib19] Soffer, N., Zaneveld, J. & Vega Thurber, R. Phage-bacteria network analysis and its implication for the understanding of coral disease. Environ. Microbiol. 17, 1203–1218 (2015). 25039472 10.1111/1462-2920.12553

[bib20] Columpsi P. et al. Beyond the gut bacterial microbiota: the gut virome. J. Med. Virol. (e-pub ahead of print 25 February 2016; doi:10.1002/jmv.24508). 10.1002/jmv.24508 PMC7166815 26919534

[bib21] Barr, J. J. et al. Bacteriophage adhering to mucus provide a non-host-derived immunity. Proc. Natl Acad. Sci. USA 110, 10771–10776 (2013). 23690590 10.1073/pnas.1305923110 PMC3696810

[bib22] Barr, J. J. et al. Subdiffusive motion of bacteriophage in mucosal surfaces increases the frequency of bacterial encounters. Proc. Natl Acad. Sci. USA 112, 13675–13680 (2015). 26483471 10.1073/pnas.1508355112 PMC4640763

[bib23] Barr, J. J., Youle, M. & Rohwer, F. Innate and acquired bacteriophage-mediated immunity. Bacteriophage 3, e258571–e258576 (2013). 10.4161/bact.25857 PMC3821666 24228227

[bib24] Angly, F. E. et al. The marine viromes of four oceanic regions. PLoS Biol. 4, 2121–2131 (2006). 10.1371/journal.pbio.0040368 PMC1634881 17090214

[bib25] Reyes, A. et al. Viruses in the faecal microbiota of monozygotic twins and their mothers. Nature 466, 334–338 (2010). 20631792 10.1038/nature09199 PMC2919852

[bib26] Johansson, M. E. V., Larsson, J. M. H. & Hansson, G. C. The two mucus layers of colon are organized by the MUC2 mucin, whereas the outer layer is a legislator of host—microbial interactions. Proc. Natl Acad. Sci. USA 108, 4659–4665 (2011). 20615996 10.1073/pnas.1006451107 PMC3063600

[bib27] Earle, K. A. et al. Quantitative imaging of gut microbiota spatial organization. Cell Host Microbe 18, 478–488 (2015). 26439864 10.1016/j.chom.2015.09.002 PMC4628835

[bib28] Johansson, M. E. V. et al. The inner of the two Muc2 mucin-dependent mucus layers in colon is devoid of bacteria. Proc. Natl Acad. Sci. USA 105, 15064–15069 (2008). 18806221 10.1073/pnas.0803124105 PMC2567493

[bib29] Tu, Q. V., Mcguckin, M. A. & Mendz, G. L. *Campylobacter jejuni* response to human mucin MUC2: modulation of colonization and pathogenicity determinants. J. Med. Microbiol. 57, 795–802 (2008). 18566135 10.1099/jmm.0.47752-0

[bib30] Kawakubo, M. et al. Natural antibiotic function of a human gastric mucin against *Helicobacter pylori* infection. Science 305, 1003–1006 (2004). 15310903 10.1126/science.1099250

[bib31] Quinn, R. A. et al. Biogeochemical forces shape the composition and physiology of polymicrobial communities in the cystic fibrosis lung. MBio 5, 1–13 (2014). 10.1128/mBio.00956-13 PMC3967525 24643867

[bib32] Kragh, K. N. et al. Role of multicellular aggregates in biofilm formation. MBio 7, 1–11 (2016). 10.1128/mBio.00237-16 PMC4807362 27006463

[bib33] Matsui H. et al. A physical linkage between cystic fibrosis airway surface dehydration and Pseudomonas aeruginosa biofilms. Proc. Natl Acad. Sci. USA 103: 18131–18136. (2006). 10.1073/pnas.0606428103 PMC1838718 17116883

[bib34] Kummerli R., Griffin A. S., West S. A., Buckling A. & Harrison F. Viscous medium promotes cooperation in the pathogenic bacterium *Pseudomonas aeruginosa*. Proc. R. Soc. B Biol. Sci. 2009; 276: 3531–3538. 10.1098/rspb.2009.0861 PMC2817189 19605393

[bib35] Cone, R. A. Barrier properties of mucus. Adv. Drug Deliv. Rev. 61, 75–85 (2009). 19135107 10.1016/j.addr.2008.09.008

[bib36] Stern, A. & Sorek, R. The phage-host arms race: Shaping the evolution of microbes. Bioessays 33, 43–51 (2010). 10.1002/bies.201000071 PMC3274958 20979102

[bib37] Avrani, S. & Lindell, D. Convergent evolution toward an improved growth rate and a reduced resistance range in *Prochlorococcus* strains resistant to phage. Proc. Natl Acad. Sci. USA 112, e2191–e2200 (2015). 25922520 10.1073/pnas.1420347112 PMC4418883

[bib38] Våge, S., Storesund, J. E. & Thingstad, T. F. Adding a cost of resistance description extends the ability of virus-host model to explain observed patterns in structure and function of pelagic microbial communities. Environ. Microbiol. 15, 1842–1852 (2013). 23331773 10.1111/1462-2920.12077

[bib39] Lynch, M. & Marinov, G. K. The bioenergetic costs of a gene. Proc. Natl Acad. Sci. USA 112, 15690–15695 (2015). 26575626 10.1073/pnas.1514974112 PMC4697398

[bib40] Bondy-Denomy, J. & Davidson, A. R. When a virus is not a parasite: the beneficial effects of prophages on bacterial fitness. J. Microbiol. 52, 235–242 (2014). 24585054 10.1007/s12275-014-4083-3

[bib41] Paterson, S. et al. Antagonistic coevolution accelerates molecular evolution. Nature 464, 275–278 (2010). 20182425 10.1038/nature08798 PMC3717453

[bib42] Bobay, L., Touchon, M. & Rocha, E. P. C. Pervasive domestication of defective prophages by bacteria. Proc. Natl Acad. Sci. USA 111, 12127–12132 (2014). 25092302 10.1073/pnas.1405336111 PMC4143005

[bib43] Brockhurst, M. A., Morgan, D. & Rainey, P. B. & Buckling, A. Population mixing accelerates coevolution. Ecol. Lett. 58, 975–979 (2003).

[bib44] Schrag, S. J. & Mittler, J. E. Host-parasite coexistence: the role of spatial refuges in stabilizing bacteria-phage interactions. Am. Nat. 148, 348–377 (1996).

[bib45] Klimenko, A. I., Matushkin, Y. G., Kolchanov, N. A. & Lashin, S. A. Bacteriophages affect evolution of bacterial communities in spatially distributed habitats: a simulation study. BMC Microbiol. 16, 31. 26823184 10.1186/s12866-015-0620-4 PMC4895265

[bib46] Peters, B. M., Jabra-rizk, M. A., Costerton, J. W. & Shirtliff, M. E. Polymicrobial interactions: impact on pathogenesis and human disease. Clin. Microbiol. Rev. 25, 193–213 (2012). 22232376 10.1128/CMR.00013-11 PMC3255964

[bib47] Sato, Y., Civiello, M., Bell, S. C., Willis, B. L. & Bourne, D. G. Integrated approach to understanding the onset and pathogenesis of black band disease in corals. Environ. Microbiol. 18, 752–765 (2016). 26549807 10.1111/1462-2920.13122

[bib48] Quinton, P. M. Chloride impermeability in cystic fibrosis. Nature 301, 421–422 (1983). 6823316 10.1038/301421a0

[bib49] Burgel, P.-R., Montani, D., Danel, C., Dusser, D. J. & Nadel, J. A. A morphometric study of mucins and small airway plugging in cystic fibrosis. Thorax 62, 153–161 (2007). 16928707 10.1136/thx.2006.062190 PMC2111259

[bib50] Lim, Y. W. et al. Mechanistic Model of *Rothia mucilaginosa* Adaptation toward Persistence in the CF Lung, Based on a Genome Reconstructed from Metagenomic Data. PLoS ONE 8, e64285 (2013). 23737977 10.1371/journal.pone.0064285 PMC3667864

[bib51] Willner, D. et al. Spatial distribution of microbial communities in the cystic fibrosis lung. ISME J. 6, 471–474 (2011). 21796216 10.1038/ismej.2011.104 PMC3260497

[bib52] Behrends, V. et al. Metabolic adaptations of *Pseudomonas aeruginosa* during cystic fibrosis chronic lung infections. Environ. Microbiol. 15, 398–408 (2013). 22882524 10.1111/j.1462-2920.2012.02840.x

[bib53] Willner, D. et al. Case studies of the spatial heterogeneity of DNA viruses in the cystic fibrosis lung. Am. J. Respir. Cell Mol. Biol. 46, 127–131 (2012). 21980056 10.1165/rcmb.2011-0253OC PMC3361360

[bib54] Lim, Y. W. et al. Metagenomics and metatranscriptomics: Windows on CF-associated viral and microbial communities. J. Cyst. Fibros. 12, 154–164 (2013). 22951208 10.1016/j.jcf.2012.07.009 PMC3534838

[bib55] James, C. E. et al. Lytic activity by temperate phages of *Pseudomonas aeruginosa* in long-term cystic fibrosis chronic lung infections. ISME J. 9, 1391–1398 (2014). 25461970 10.1038/ismej.2014.223 PMC4351911

[bib56] Davies, E. V. et al. Temperate phages enhance pathogen fitness in chronic lung infection. ISME J. (e-pub ahead of print 12 April 2016; doi:10.1038/ismej.2016.51). 10.1038/ismej.2016.51 PMC4950967 27070941

[bib57] Winstanley, C. et al. Newly introduced genomic prophage islands are critical determinants of in vivo competitiveness in the Liverpool Epidemic Strain of *Pseudomonas aeruginosa*. Genome Res. 1, 12–23 (2009). 10.1101/gr.086082.108 PMC2612960 19047519

[bib58] Ratjen, F., Döring, G. & Nikolaizik, W. H. Effect of inhaled tobramycin on early *Pseudomonas aeruginosa* colonisation in patients with cystic fibrosis. Lancet 358, 983–984 (2001). 11583754 10.1016/S0140-6736(01)06124-4

[bib59] Han, M. K. et al. Significance of the microbiome in obstructive lung disease. Thorax 67, 456–464 (2012). 22318161 10.1136/thoraxjnl-2011-201183 PMC3578398

[bib60] Smyth, A. Prophylactic antibiotics in cystic fibrosis: a conviction without evidence? Pediatr. Pulmonol. 40, 471–476 (2005). 16200640 10.1002/ppul.20305

[bib61] Stutman, H. R., Lieberman, J. M., Nussbaum, E. & Marks, M. I. Antibiotic prophylaxis in infants and young children with cystic fibrosis: A randomized controlled trial. J. Pediatr. 140, 299–305 (2002). 11953726 10.1067/mpd.2002.121930

[bib62] Rolain, J. M., Fancello, L., Desnues, C. & Raoult, D. Bacteriophages as vehicles of the resistome in cystic fibrosis. J. Antimicrob. Chemother. 66, 2444–2447 (2011). 21816766 10.1093/jac/dkr318

[bib63] Stevens, D. L. et al. Impact of antibiotics on expression of virulence- associated exotoxin genes in methicillin-sensitive and methicillin-resistant *Staphylococcus aureus*. J. Infect. Dis. 175, 202–211 (2007). 10.1086/510396 17191165

[bib64] Maiques, E. et al. Beta-lactam antibiotics induce the SOS response and horizontal transfer of virulence factors in *Staphylococcus aureus*. J. Bacteriol. 188, 2726–2729 (2006). 16547063 10.1128/JB.188.7.2726-2729.2006 PMC1428414

[bib65] Shapiro, O. H., Kramarsky-winter, E., Gavish, A. R., Stocker, R. & Vardi, A. A coral-on-a-chip microfluidic platform enabling live-imaging microscopy of reef-building corals. Nat. Commun. 7, 1–9 (2016). 10.1038/ncomms10860 PMC4785229 26940983

[bib66] Drescher, K. et al. Architectural transitions in *Vibrio cholerae* biofilms at single-cell resolution. Proc. Natl Acad. Sci. USA 113, E2066–E2072 (2016). 26933214 10.1073/pnas.1601702113 PMC4833255

[bib67] Zhang, H. & Liu, K. Optical tweezers for single cells. J. R. Soc. Interf. 5, 671–690 (2008). 10.1098/rsif.2008.0052 PMC2408388 18381254

[bib68] Gawad, C., Koh, W. & Quake, S. Single-cell genome sequencing: current state of the science. Nat. Rev. Genet. 17, 175–188 (2016). 26806412 10.1038/nrg.2015.16

